# The gastropod shell has been co-opted to kill parasitic nematodes

**DOI:** 10.1038/s41598-017-04695-5

**Published:** 2017-07-06

**Authors:** R. Rae

**Affiliations:** Liverpool John Moores University, School of Natural Sciences and Psychology, Byrom Street, Liverpool, L33AF UK

## Abstract

Exoskeletons have evolved 18 times independently over 550 MYA and are essential for the success of the Gastropoda. The gastropod shell shows a vast array of different sizes, shapes and structures, and is made of conchiolin and calcium carbonate, which provides protection from predators and extreme environmental conditions. Here, I report that the gastropod shell has another function and has been co-opted as a defense system to encase and kill parasitic nematodes. Upon infection, cells on the inner layer of the shell adhere to the nematode cuticle, swarm over its body and fuse it to the inside of the shell. Shells of wild *Cepaea nemoralis*, *C. hortensis* and *Cornu aspersum* from around the U.K. are heavily infected with several nematode species including *Caenorhabditis elegans*. By examining conchology collections I show that nematodes are permanently fixed in shells for hundreds of years and that nematode encapsulation is a pleisomorphic trait, prevalent in both the achatinoid and non-achatinoid clades of the Stylommatophora (and slugs and shelled slugs), which diverged 90–130 MYA. Taken together, these results show that the shell also evolved to kill parasitic nematodes and this is the only example of an exoskeleton that has been co-opted as an immune system.

## Introduction

The evolution of the shell has aided in the success of the Gastropoda, which are composed of 65–80,000 species that have colonised terrestrial and marine environments over 400MY^1, 2^. The gastropod shell shows a vast array of different sizes, shapes and structures, and is made of an outer proteinaceous periostracum of conchiolin and sub-layers of crystalline calcium carbonate^3^. The shell allows protection from predators but slugs and snails are also frequently attacked by parasitic flies, mites, trematodes and nematodes^4–6^. Of these the nematodes are the most abundant with 108 mollusc parasitic species present in four out of the five clades of the Nematoda^7, 8^. Gastropods are used by nematodes as definitive, intermediate, necromenic or phoretic hosts. For example, *Caenorhabditis elegans* uses gastropods for transport^9^; *Angiostrongylus cantonensis*, the causal agent of human eosinophilic meningoencephalitis, uses snails as intermediate hosts^10^ and *Phasmarhabditis hermaphrodita* can kill several species of slugs and snails^11, 12^. Because of its pathogenic nature this nematode has been formulated into a biological control agent (Nemaslug®) by BASF-UK for use by farmers and gardeners^11, 12^. Nematodes are applied to soil, hunt for slugs which they infect and kill 4–21 days later^11, 13^. Pestiferous slugs from the family Agriolimacidae are particularly susceptible to *P. hermaphrodita* whereas many snail species are largely resistant for reasons unknown^11, 14^. The major anatomical difference between slugs and snails is the presence of the snail’s shell, unlike slugs, which have a reduced internalised shell^5^. Recent studies have shown that upon nematode infection some snail species (*Lissachatina fulica* and *Cepaea nemoralis*) have nematodes trapped in their shells^15, 16^ but this process is remarkably uncharacterised and not understood. It is unknown how common or evolutionarily conserved this response is; whether it is specific to one nematode species or whether this is a laboratory based phenomenon or is a common procedure used in the wild by snails to kill parasitic nematodes. Furthermore, there is no information on what species of parasitic nematodes are encased and killed in wild caught snail shells and for how long they can remain in shells. Ultimately, this research could unravel a new role for the gastropod shell and reveal that exoskeletons can evolve new immunological abilities to provide protection against parasites such as nematodes.

## *C. nemoralis* under lab conditions can trap, encase and kill parasitic nematodes

**Figure 1:**
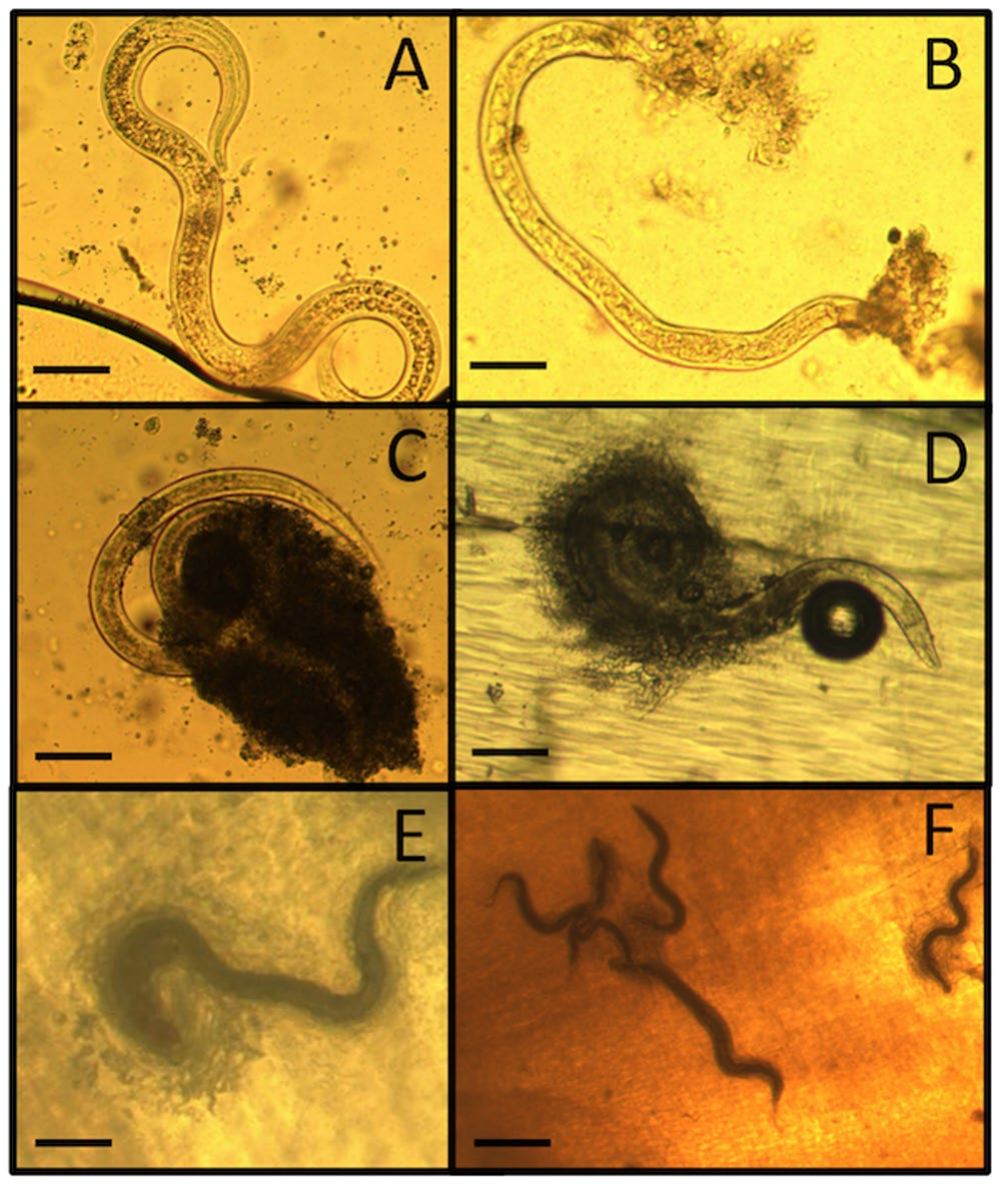
Nematodes can be trapped and killed in the shell of *C. nemoralis*. *P. hermaphrodita* (**A**) can be encapsulated in the shell of *C. nemoralis*. After 1 (**B**) and 3 days (**C**) cells attach to the nematode cuticle. The numbers of cells increases over time engulfing the nematode body and then it adheres to the inner snail shell (**D**). The cells continue to cover the entire body of the nematode and it becomes completely encased and fused to the inner layer of the shell (**E**,**F**). Scale bars represent 100 μm.

To investigate the role of the shell as a defence mechanism against nematodes *C. nemoralis* (a resistant species)^14, 17^ were exposed to two wild strains of *P. hermaphrodita* (C11 and C25). After 1 and 3 days of infection the snails were dissected and surprisingly, the nematodes were found attached to the inner shell surface (Fig. 1)^16^. Over time these cells multiplied on the inside of the shell, attached to the nematode cuticle and swarmed over the entire body and engulfed it (Fig. 1). When the nematodes were completely covered they were then fused to the inner layer of the shell.

**Figure 2:**
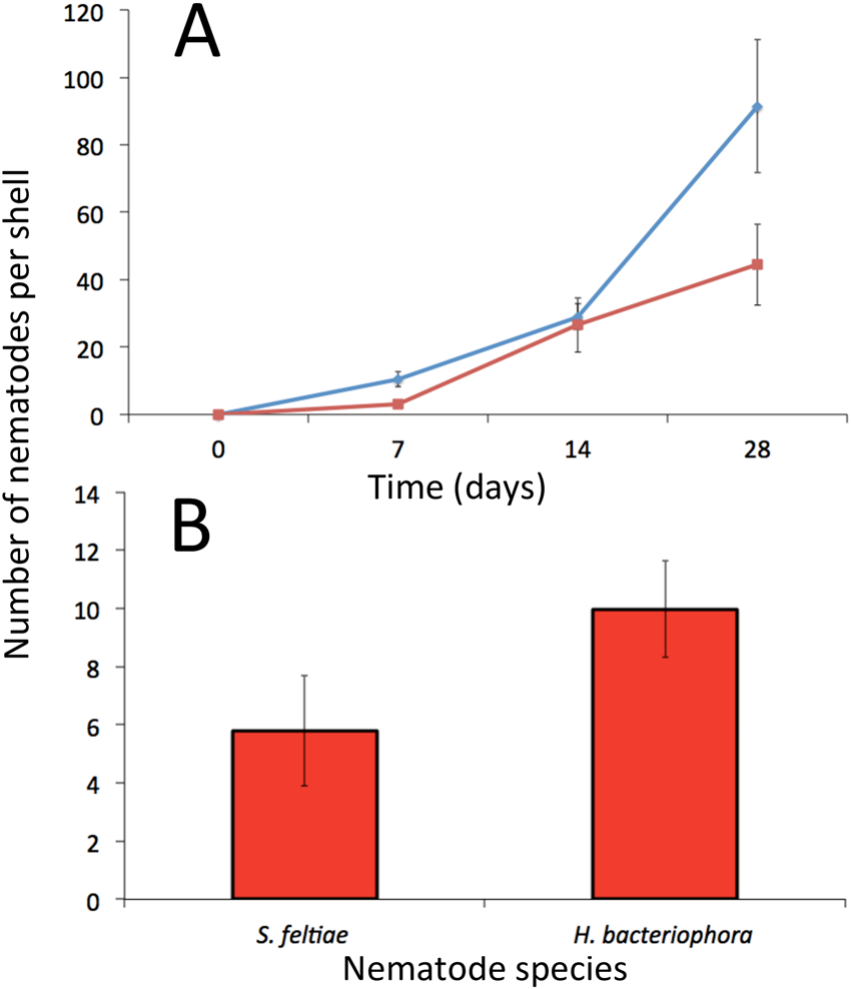
*C. nemoralis* can trap and kill different nematode species. Mean number of *P. hermaphrodita* C11 (blue) and C25 (red) found encased in *C. nemoralis* shells after 7, 14 and 28 days infection (**A**) (n = 10 *C. nemoralis* for each time point exposed to each strain). Mean number of *S. feltiae* and *H. bacteriophora* in *C. nemoralis* shells (n = 10 and n = 30, respectively) after 7 days exposure (**B**). Bars represent ± one standard error.

The numbers of *P. hermaphrodita* (both strain C25 and C11) that were encapsulated increased significantly from day 7 to 28 (P < 0.01, Fig. 2A). The engulfment of foreign bodies is a powerful way to combat parasites and has evolved in eukaryotes from amoebae to vertebrates^18^. A similar process may allow *C. nemoralis* to combat infection by pathogenic nematodes by trapping and killing them using their shell.

In order to understand if this cellular process was specific to just *P. hermaphrodita* the experiment was repeated with distantly related nematodes (*Steinernema feltiae* and *Heterorhabditis bacteriophora*). These nematodes are used as biological control agents of insects that vector toxic bacteria (*Xenorhabdus* or *Photorhabdus* spp.) into the insect haemocoel and cause death in 24–48 hours^19^. They are not pathogenic to terrestrial gastropods^20^ but were also trapped, encased and killed in *C. nemoralis* shells (Fig. 2B). There was no significant difference between the numbers of *S. feltiae* and *H. bacteriophora* trapped in the shells of *C. nemoralis* after 7 days (P = 0.09). Thus, the ability of the shell to encapsulate nematodes has not evolved to specifically kill slug parasitic nematodes but is a general process that can kill several diverse parasitic nematodes.

## Snails collected from the wild have nematodes encased in their shells

**Figure 3:**
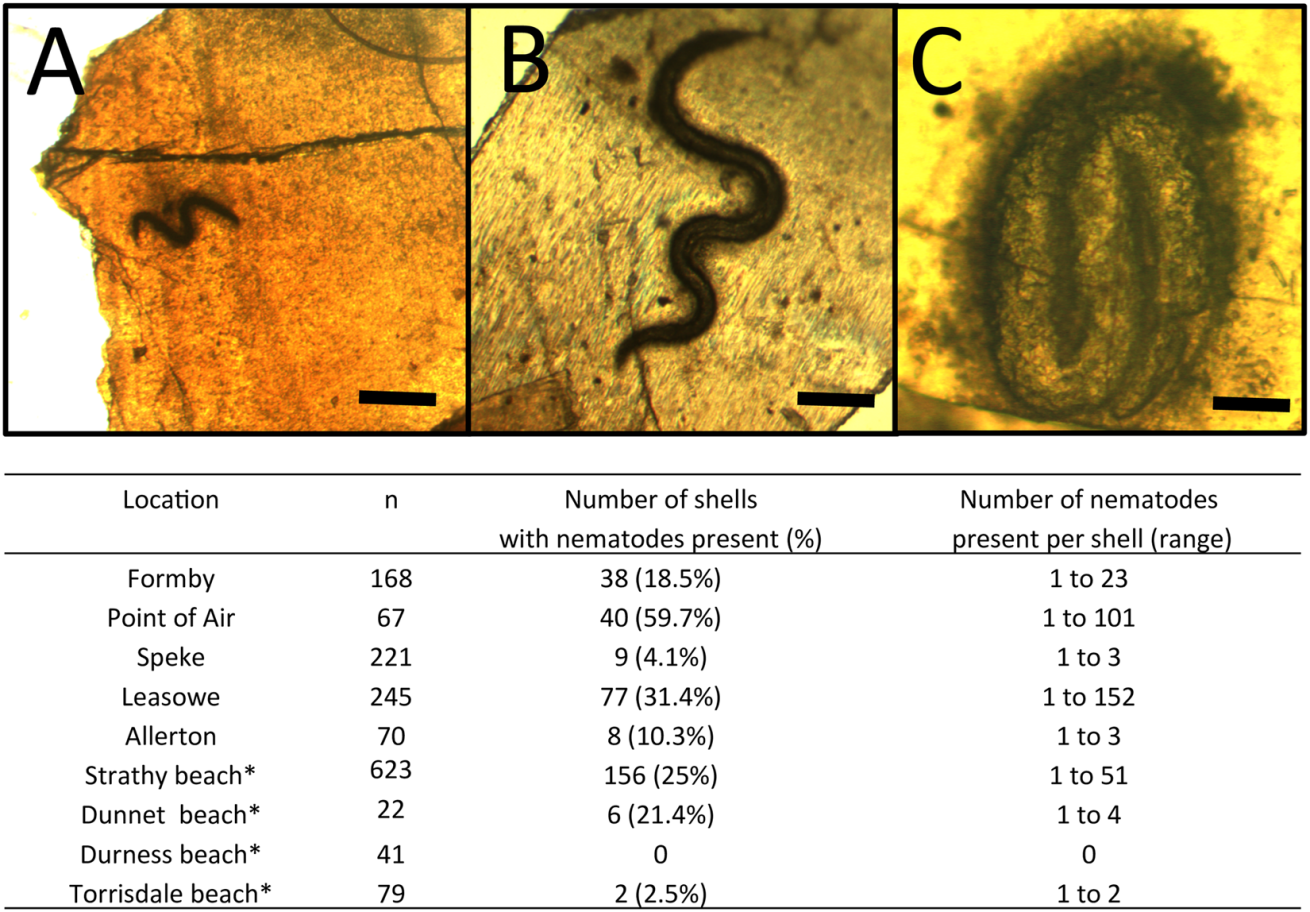
Wild caught snails encase nematodes in their shells. *C. nemoralis* and *C. hortensis* from around the U.K have many nematodes present in the inner aperture of their shells (**A**–**C**). The table below details the total numbers of *C. nemoralis* and *C. hortensis* shells examined. *C. hortensis* were collected from locations marked with *. Scale bars in (**A**,**B** and **C**) represent 500 μm, 1 cm and 100 μm, respectively.

Under laboratory conditions *C. nemoralis* can encapsulate and kill parasitic nematodes but does this process occur in nature? Shells of *C. nemoralis* and its congener (*C. hortensis*) were collected from sand dunes in north west England and the north of Scotland, respectively (Figure S1, Table S1) and examined for presence of nematodes. Nematodes were found fixed in shells of both wild *C. nemoralis* and *C. hortensis* shells (Fig. 3A–C). The numbers of nematodes found in *C. nemoralis* shells collected from several locations in north west England and *C. hortensis* shells from northern Scotland differed significantly (*C. nemoralis* ${\mathscr{X}}$_2_ (4) = 93.06, P < 0.0001 and *C. hortensis χ*_2_ (3) = 430.54, P < 0.0001). In some cases 60% of the shells collected had nematodes present in numbers ranging from 1 to 101 nematodes per shell. Also *Cornu aspersum* shells purchased from an Escargot farm in the U.K. had nematodes present within 100% of shells (n = 136), having a mean of 31 ± 2 nematodes per shell. Thus, several snail species isolated from the wild can encase and kill nematodes.

## Nematode DNA can be amplified for species identification from shells

In order to understand what nematode species were naturally infecting these shells, they were homogenized and using PCR and DNA sequencing of the nuclear Internal Transcribed Spacer gene (ITS) of several *C. nemoralis* shells and 16 *C. aspersum* shells, the nematodes present in *C. nemoralis* were identified as *P. hermaphrodita*, *Phasmarhabditis californica*, *Eucephalobus* sp. and, based on morphology alone, a Mermithid sp. (Fig. 3B). All *C. aspersum* shells were found to have *C. elegans*, a nematode associated with slugs and snails^21^, present in great numbers encased in their shells. Therefore, wild caught and farmed snails frequently use their shell to kill a diverse range of nematode species.

## Nematodes are present in the shells of museum collections >500 years old

Next, to understand whether nematodes were permanently fixed in shells when killed, I examined collections of *C. nemoralis* housed in Liverpool and Manchester museums, a large part of which contains Arthur Cain’s U.K. wide survey of *C. nemoralis* from 1950^22^. From 1,406 *C. nemoralis* shells, specimens collected in 1960, 1918, 1909, 1866 and even 1864 had nematodes encased in their shells (Figure S2, Table S2). Also sub-fossil snail shells of *C. aspersum, C. nemoralis* and *C. hortensis*, estimated to be >500 years old^23^, were also examined and nematodes were found to be present, albeit in low amounts (n = 76 shells, 1 *C. nemoralis* shell had 2 nematodes present, Figure S3). Therefore, when nematodes are encapsulated they are fixed for hundreds of years as a permanent record of parasitism, which permits an evolutionary analysis of shells across the Stylommatophora.

## Parasitic nematode encapsulation is evolutionary conserved across the Stylommatophora

**Figure 4:**
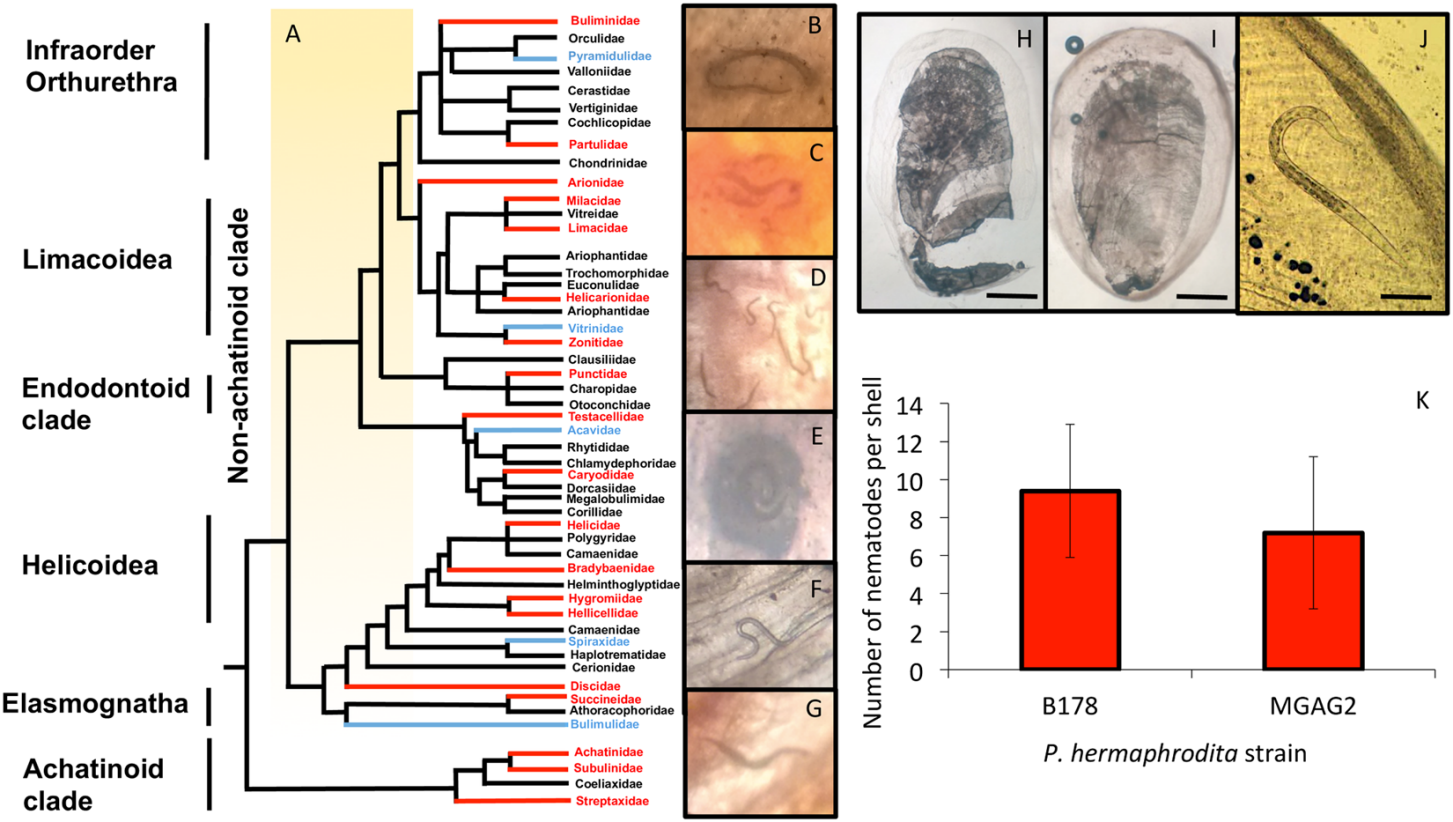
Nematode encapsulation is evolutionary conserved across the Stylommatophora. The evolutionary relationship between members of the Stylommatophora has been debated for over 100 years^35^. A representative molecular phylogeny^5^ which is in agreement with morphological classifications^36^ was used (**A**). Shells from families had nematodes present (red) or absent (blue) or not examined (black). For exact number of shells examined and numbers of nematodes present see Table S2. Nematodes were found in the shells of the following snails species: *Partula rosea* (**B**), *Gudeoconcha sophiae* (**C**), *C. aspersum* (**D**), *Helicostyla intorta* (**E**), *Succinea putris* (**F**) and *Glessula inornata* (**G**). As well as snails, slugs (*D. panormitanum*) were shown to encapsulate and kill parasitic nematodes in their internal shell. Uninfected *D. panormitanum* (**H**) do not have nematodes present but when infected with two *P. hermaphrodita* strains (B178 and MGAG2) nematodes are found encased in the thick gelatinous membrane of the internal shell (**I** and **J**) and were quantified (**K**). Scale bars in H and I represent 1 mm and in J represents 100 μm.

Terrestrial snails and slugs are members of the Stylommatophora, which consists of 60–85,000 species split into two major clades known as the ‘achatinoid’ clade (Achatinidae, Subulinidae and Streptaxidae) and the ‘non-achatinoid’ clade (Limacoidea, Orthurethra, Helicoidea and the Elasmognatha)^5^. To understand how evolutionary conserved encapsulation of nematodes is across the Stylommatophora I examined 1,321 individual shells of 43 genera and 20 families that had been collected from around the world by great conchologists such as John Jackson, Albert Salisbury and Arthur Stelfox from the late 1800’s to early to mid 1900’s. Nematodes were found encapsulated in 28 species from 12 different families across all 4 infraorders of snails in the achatinoid clade and in 6 species from all 3 families of the non-achatinoid clade^5^ (Fig. 4A–F, Table S3). Therefore, the ability to trap, encase and kill nematodes is a pleisomorphic trait that would have been present in the ancestor of the achatinoid and non- achatinoid clades, which was present before their diversification 90–130 MYA^24, 25^.

## Slugs also can trap, encase and kill nematodes in their reduced shell

As well as snails, slugs have evolved several times throughout the Stylommatophora^6^. The main anatomical difference between them is that the shell in slugs has become reduced and has been internalized in most species^6, 26^. Slugs (*Deroceras panoramitanum*) were also shown to trap and kill nematodes in their shell. *D. panormitanum* were infected separately with two strains of *P. hermaphrodita* (MGAG2 and B178) and after 28 days the numbers of nematodes in the shell were quantified. *P. hermaphrodita* were found to be fixed in the outer layers of the internalized shell of *D. panormitanum* (Fig. 4G–J) with no significant difference between the numbers of each *P. hermaphrodita* strain found in the shells (P = 0.34). In contrast to *D. panormitanum* the shells of slugs from the genus *Testacella* are reduced in size and have not been internalized but remain external at the posterior of the slug^26^. By screening through 28 *Testacella sculutum* and 32 *T. maugei* shells that had been collected in 1910 four shells were found that had between 1 and 8 nematodes present per shell (Figure S4). Furthermore, the internalized, granulated shell particles of *Arion ater* and *A. subfuscus*, have also been observed to adhere to nematodes^14^. Thus, the slug shell, whether reduced, internalized or granulized, posing no protection against predators or abiotic factors, has retained its ability to kill parasitic nematodes.

## Discussion

These results suggest that the terrestrial gastropod shell is an exaptation that has been co-opted as a defense system to combat parasitic nematodes. Co-option is essential for the production of new physiological, biochemical and morphological processes^27^ but there are only a handful of examples of the co-option of morphological features. For example, feathers were co-opted for flight and originally used for communication using light and colour in dinosaurs^28^ and the shell of turtles initially aided digging, not protection^29^. There are no examples of morphological structures being co-opted with new immunological roles.

Nematodes and gastropods have been engaged in a co-evolutionary arms race since the appearance of gastropods in the late Cambrian^5, 7, 8^. The shell seems to be a formidable defence system that is able to quickly trap hundreds of nematodes. It is unknown how cells of the shell recognise and attach to the nematode cuticle, but they could respond to lectins, mucins, glycoproteins or collagens that are present on the nematode surface coat and cuticle^30^. However, some nematodes, such as *P. hermaphrodita*, have evolved ways to evade capture as several snail species e.g. *Cernuella virgata* are susceptible to infection and can be killed by this nematode^12, 14–16^. The precise mechanism of how they can evade the shell is unknown but animal, plant and free-living nematodes display antigenic variation of their surface coat^31^. These antigens can be split into two groups including somatic antigens and excretory/secretory (ES) antigens, which are released from the parasite during infection and play various roles in parasitism and immune responses of hosts^32^. A similar process may be used by gastropod parasitic nematodes to evade the shells capture.

This encapsulation ability seems restricted to land snails, but the molluscan shell may be underrated as a defence mechanism against metazoan parasites. For example, pearls from bivalves are thought to be created by encapsulation of trematodes and foreign material in the shell^33^. However, the research presented here shows that encapsulation is a nematode specific process as from ~5,000 shells examined there were no traces of other metazoan parasites that infect gastropods such as trematodes or parasitic flies found^6^. This is presumably because trematodes have evolved to use snails as intermediate hosts, and thus do not adversely affect them, and parasitic flies would be too large to encapsulate.

As the struggle against infectious agents can lead to rapid changes in the immune system^34^ terrestrial gastropods were able to co-opt the use of their shell not just as protection against predators but also to combat nematode parasites 90–130 MYA^24, 25^. This co-opted ability shows that biological armour can evolve novel defense mechanisms. This ability has contributed to the success of molluscs in colonising all ecological niches of Earth for hundreds of millions of years.

## Methods

### Infection assays with *P. hermaphrodita*

*P. hermaphrodita* were isolated from slugs (*Limax flavus* and *D. panormitanum*) from Sefton Park, Liverpool. The nematodes were identified via standard genotyping procedures^8^. *P. hermaphrodita* were grown on rotting *L. flavus* to the dauer stage^13^ and exposed to *C. nemoralis* for 28 days^11, 14^. Plastic non-airtight boxes (10 × 10 cm) were half-filled with 25 g soil. *P. hermaphrodita* were applied at the field application rate of 30 per cm^2^ ^11^. Both *S. feltiae* and *H. bacteriophora* were also applied at the recommended field application rate (50 nematodes per cm^2^). Five *C. nemoralis* or *D. panormitanum* were added to each box and were fed cucumber every 3–4 days. After 7, 14 and 28 days the snails were dissected and the numbers of nematodes in their shells quantified. These times points were chosen as they were shown in previous studies^14–16^ to be of sufficient time for nematodes to penetrate and infect both slugs and snails.

### Molecular identification of nematodes in snail shells

Shells of *C. aspersum* were homogenized using sterile plastic Eppendorf pestles and DNA was then extracted using a Qiagen DNA extraction kit. Individual nematodes in *C. nemoralis* shells were extracted using sterile scalpel blades. DNA was extracted using GeneJET Genomic DNA purification kit (Thermo Fisher Scientific) and the ITS gene was amplified using the primer pair N93 5′-TTGAACCGGGTAAAAGTCG-3′ and N94 5′-TTAGTTTCTTTTCCTCCGCT-3′. PCR conditions were: 2 mins at 95 °C, followed by 35 cycles of 15 s at 95 °C, 15 s at 50 °C and 2 mins at 72 °C and finally 7 mins at 72 °C. PCR products were then purified using GeneJET PCR purification kit (Thermo Fisher Scientific). The PCR product was sequenced in forward and reverse directions using the same primers previously described and the sequence was compared with GenBank sequences using BLASTN searches.

### Statistical analysis

The numbers of *P. hermaphrodita* (C11 and C25) that were found in the shells of *C. nemoralis* over time were analysed using a One-way ANOVA and Tukey’s post hoc test. The numbers of *S. feltiae* and *H. bacteriophora* that were found in the shells of *C. nemoralis* as well as the numbers of *P. hermaphrodita* (MGAG2 and B178) found infecting *D. panormitanum* were compared using a Student’s t test. The numbers of unknown nematodes that were found in *C. nemoralis* and *C. hortensis* shells collected from around the U.K. were compared using a Chi squared test.

## Electronic supplementary material

Supplementary Figures 1–4

Supplementary Tables 1–3

## References

[1] Porter SM (2007). Seawater chemistry and early carbonate biomineralization. Science.

[2] Barker, G. M. *Biology of Terrestrial molluscs* (CABI Publishing, 2001).

[3] Lowenstam, H. A. & Weiner, S. *On Biomineralization*. (Oxford University Press, New York, 1989).

[4] Vermeij, G. J. *A Natural History of Shells* (Princeton Univ. Press, 1993).

[5] Wade CM, Mordan PB, Clarke B (2001). A phylogeny of the land snails (Gastropoda: Pulmonata). Proc. R. Soc. Lond. B..

[6] Barker, G. M. *Natural Enemies of Terrestrial Molluscs* (CABI Publishing, U.K., 2004).

[7] Grewal PS, Grewal SK, Tan L, Adams BJ (2003). Parasitism of molluscs by nematodes: types of associations and evolutionary trends. J. Nematol..

[8] Blaxter ML (1998). A molecular evolutionary framework for the phylum Nematoda. Nature.

[9] Petersen C (2015). Travelling at a slug’s pace: possible invertebrate vectors of *Caenorhabditis* nematodes. BMC Ecology..

[10] Wang QP, De-Hua L, Xing-Quan Z, Xiao-Guang C, Zhao-Rong L (2007). Human angiostrongyliasis. Lancet Infect. Dis..

[11] Wilson MJ, Glen DM, George SK (1993). The rhabditid nematode *Phasmarhabditis hermaphrodita* as a potential biological control agent for slugs. Biocontrol Sci. Technol..

[12] Rae R, Verdun C, Grewal PS, Robertson JF, Wilson MJ (2007). Biological control of terrestrial molluscs using *Phasmarhabditis hermaphrodita* - progress and prospects. Pest Manag. Sci..

[13] Tan L, Grewal PS (2001). Infection behaviour of the rhabditid nematode *Phasmarhabditis hermaphrodita* to the grey garden slug *Deroceras reticulatum*. J. Parasitol..

[14] Rae RG, Robertson J, Wilson MJ (2009). Chemoattraction and host preference of the gastropod parasitic nematode *Phasmarhabditis hermaphrodita*. J. Parasitol..

[15] Williams A, Rae R (2015). Susceptibility of the Giant African snail (*Achatina fulica*) exposed to the gastropod parasitic nematode *Phasmarhabditis hermaphrodita*. J. Invertebr. Pathol..

[16] Williams A, Rae R (2016). *Cepaea nemoralis* uses its shell as a defence mechanism to trap and kill parasitic nematodes. J. Mollus. Stud..

[17] Wilson MJ, Hughes LA, Hamacher GM, Glen DM (2000). Effects of *Phasmarhabditis hermaphrodita* on non-target molluscs. Pest Manag. Sci..

[18] Chen S, Zhuchenko O, Kuspa A, Zhuchenko K (2007). Immune-like phagocyte activity in the social amoeba. Science.

[19] Forst S, Dowds B, Boemare N, Stackebrandt E (1997). *Xenorhabdus* and *Photorhabdus* spp.: bugs that kill bugs. Annu. Rev. Microbiol..

[20] Wilson MJ, Glen DM, Hughes LA, Pearce JD, Rodgers PB (1994). Laboratory tests of the potential of entomopathogenic nematodes for the control of field slugs (*Deroceras reticulatum*). J. Invertebr. Pathol..

[21] Kiontke, K. & Sudhaus, W. *Ecology of Caenorhabditis species* (WormBook: the online review of *C. elegans* biology, 2006). 10.1895/wormbook.1.37.1 PMC4780885 18050464

[22] Cain AJ (1968). Studies on *Cepaea* V. Sand dune populations of *Cepaea nemoralis*. Phil. Trans. R. Soc. B..

[23] Cain AJ, Cameron RAD, Parkin DT (1969). Ecology and variation of some helicid snails in northern Scotland. Proc. Malac. Soc. Lond..

[24] Tillier, S., Masselot, M. & Tillier, A. Phylogenic relationships of the pulmonate gastropods from rRNA sequences, and tempo and age of the Stylommatophoran radiation. In *Origin and evolutionary radiation of the* Mollusca (eds Taylor, J. D.) 267–284 (Oxford University Press, 1996).

[25] Bandel K (1991). Gastropods from brackish and fresh water of the Jurassic-Cretaceous transition (a systematic evaluation). Berliner Geowiss. Abh. A.

[26] Runham, N. W. & Hunter, P. J. *Terrestrial slugs* (Hutchinson Univ. Library, 1970).

[27] Gould SJ, Vrba ES (1982). Exaptation – a missing term in the science of form. Paleobiology..

[28] Foth C, Tischlinger H, Rauhut OWM (2014). New specimen of *Archaeopteryx* provides insights into the evolution of pennaceous feathers. Nature..

[29] Lyson TR (2016). Fossorial origin of the turtle shell. Curr. Biol..

[30] Lee, D. H. Cuticle, moulting and exsheathment. In *The biology of nematodes* (ed. Lee, D. H.) 171–210 (Taylor and Francis, 2002).

[31] Blaxter, M. L., Page, A. P. Rudin, W. & Maizels, R. M. 1992. Nematode surface coats: actively evading immunity. *Parasitol. Today* **8**, 243–246 (1992). 10.1016/0169-4758(92)90126-m 15463630

[32] Maruyama, H. & Nawa, Y. Immunology of nematode infection. In *The biology of nematodes* (eds Lee, D. H.) 457–482 (Taylor and Francis, 2002).

[33] Götting K-J (1979). Durch parasite induzierte perlbildung bei *Mytilus edulis* L. (Bivalvia). Malacologia..

[34] Frank, S.A. Immunology and evolution of infectious diseases (Princeton Univ. Press, 2002). 20821852

[35] Emberton KC (1990). Comparison of recent classifications of Stylommatophoran land-snail families, and evaluation of large-ribosomal-RNA sequencing for their phylogenies. Malacologia..

[36] Pilsbury HA (1900). On the zoological position of *Achatinella* and *Partula*. Proc. Acad. Nat. Sci. Philadelphia..

