# Supplementary material for: The gastropod shell has been co-opted to kill parasitic nematodes
Source: Sci Rep. 2017 Jul 6;7:4745. doi: 10.1038/s41598-017-04695-5 (PMC5500577; doi:10.1038/s41598-017-04695-5)
Supplement: Supplementary file 1 — Supplementary Figures 1–4 [file 41598_2017_4695_MOESM1_ESM.pdf]

The gastropod shell has been co-opted to kill parasitic nematodes

R. Rae[1]*

Fig. S1. Shells of dead *C. nemoralis* were collected from two locations in the U.K. (A) from Merseyside in north west England and the north of Wales (B) and *C. hortensis* were collected from the north of Scotland (C). Locations included: 1. Formby 2. Leasowe 3. Allerton 4. Speke 5. Point of Air 6. Durness beach 7. Torrisdale beach 8. Strathy beach and 9. Dunnett beach. GPS locations can be found in Supplementary Table 1. Maps were reproduced from Ordnance Survey map data by permission of Ordnance Survey © Crown copyright 2016.

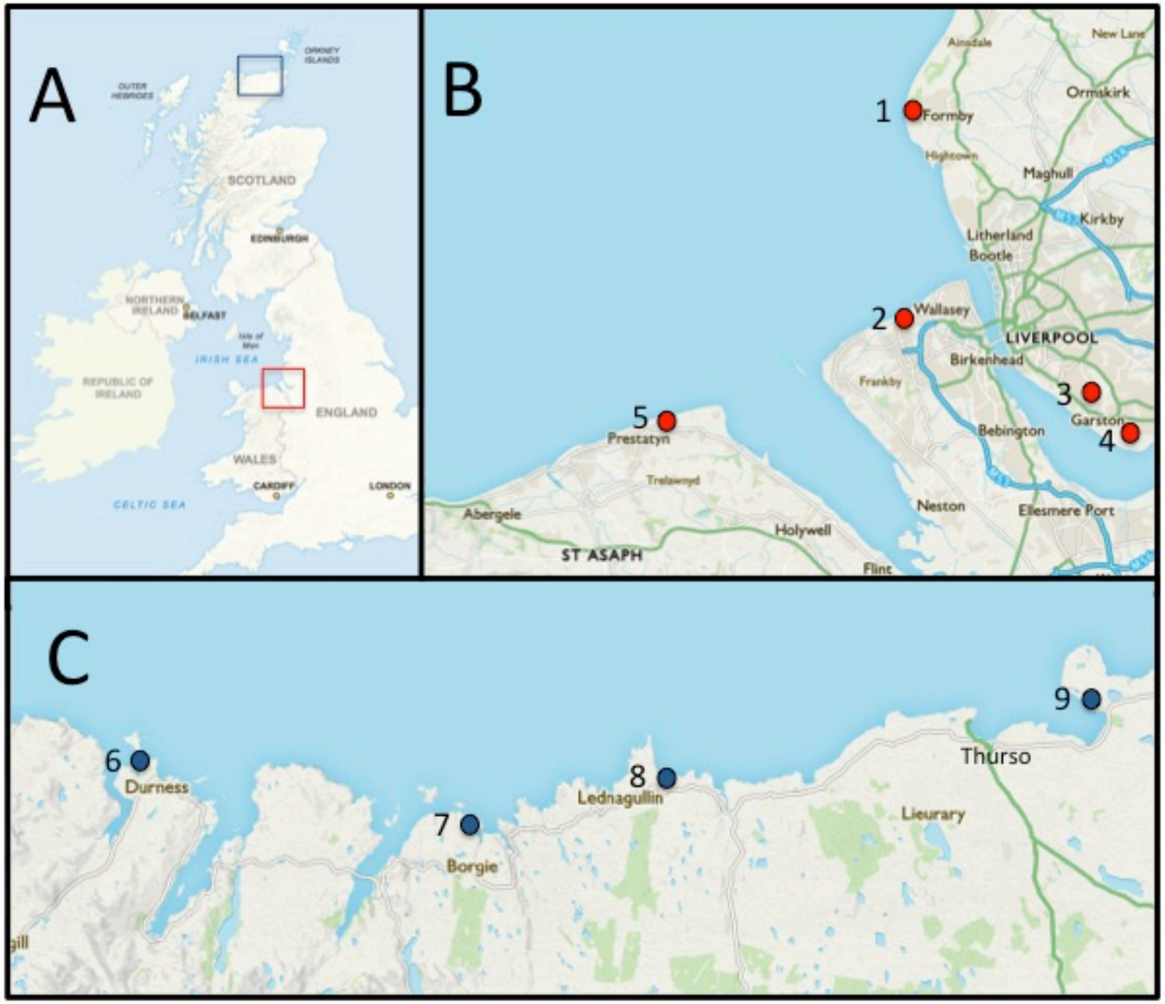

Fig. S2. Collections of *C. nemoralis* from Liverpool and Manchester museum were examined for any presence of nematodes in their shell. The table shows where and when the shells were collected. When nematodes are encased in the shells of *C. nemoralis* they are present for over 140 years as shells from 1864 (A) had nematodes present (B). Scale bar in B represents 100 µm.

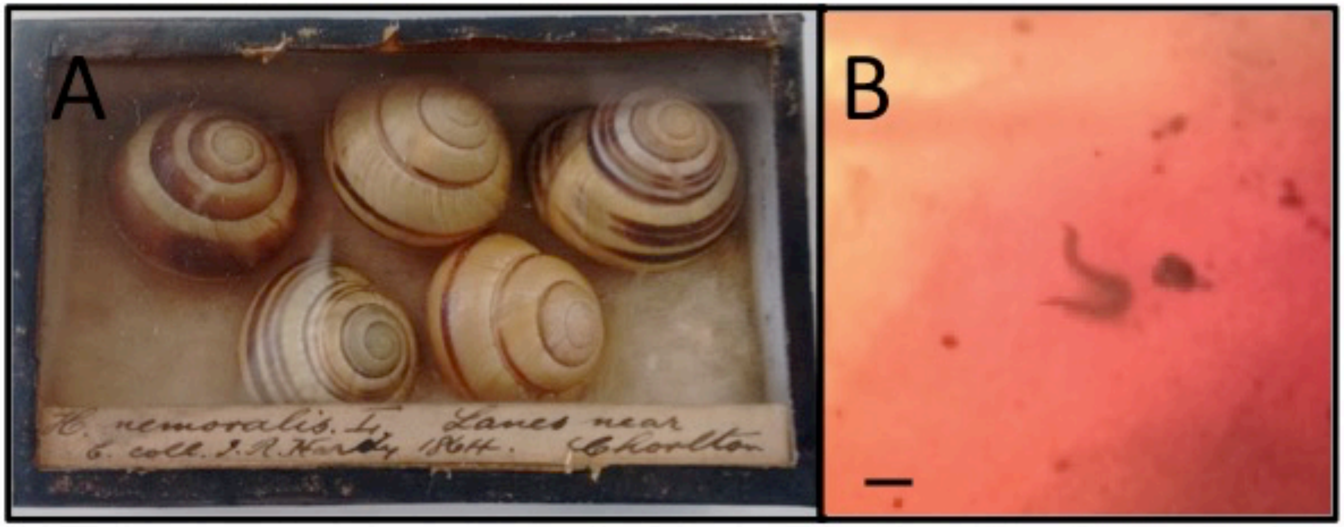

Collection location	Year	No. of shells	Shells with worms	Shells infected (%)	No. of nematodes (range)
Marlborough Down, U.K.	1963	121	8	6.6	1
Point of Ayre, Isle of man, U.K.	1962	45	15	33.3	1 to 80
Tramore Strand, Ireland	1960	100	18	18	1 to 226
Rough Down, Marlborough, U.K.	1950	87	5	5.7	1 to 1
Baguley, U.K.	1918	8	2	25	1
Mallaranny, Ireland	1909	7	2	28.6	1 to 6
Jacksons Brat, U.K.	1866	9	1	11.1	3
Chorlton, U.K.	1864	5	1	20	7

Fig. S3. Shells (*C. nemoralis, C. aspersum* and *C. hortensis*) (A) collected by Arthur Cain from the north of Scotland in 1966 are thought to be over 500 years old [23] (A). One *C. nemoralis* shell (B) had 2 nematodes present (C). Scale bar in C represent 500 µm.

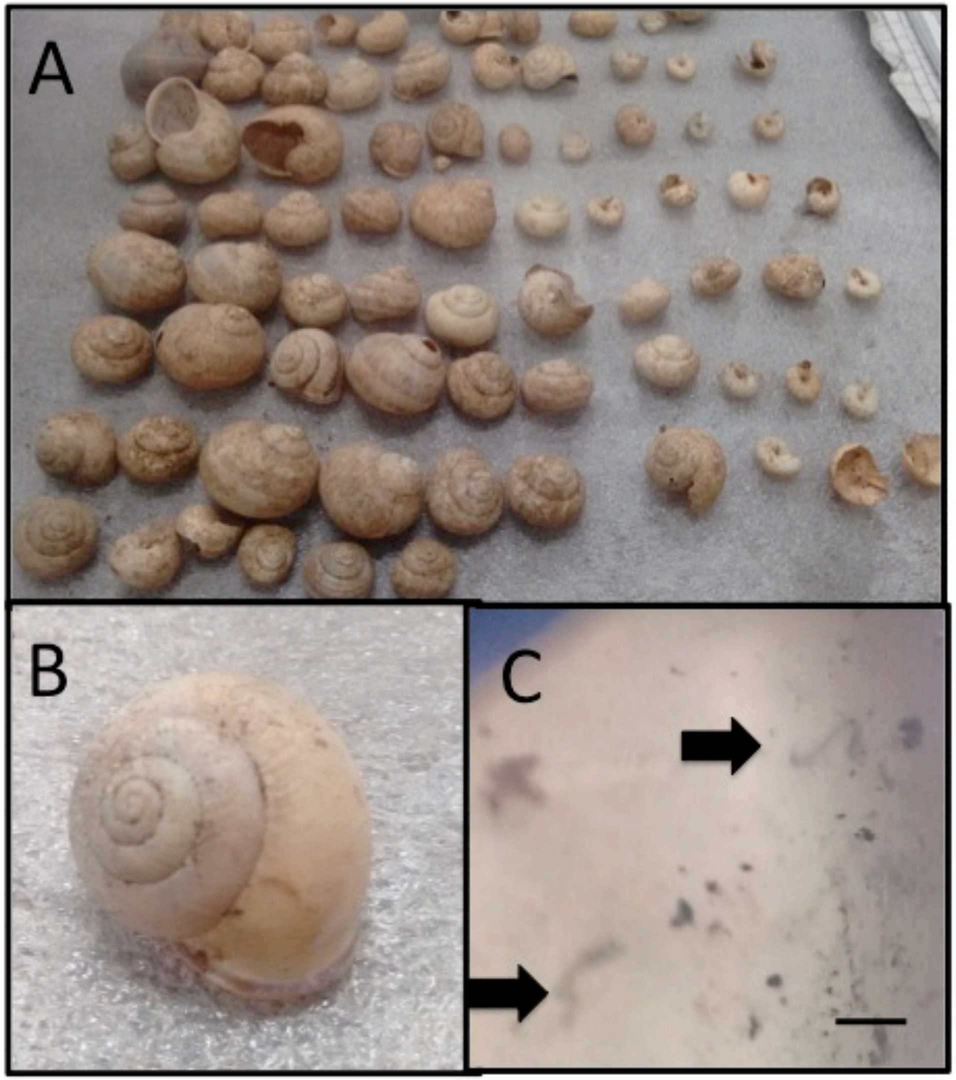

Fig. S4. Slugs from the genus *Testacella* have a reduced external shell positioned at the posterior. Shells collected from 1910 (A, B) and housed in Liverpool museum were examined for nematodes and were found to be present (C) in two species.

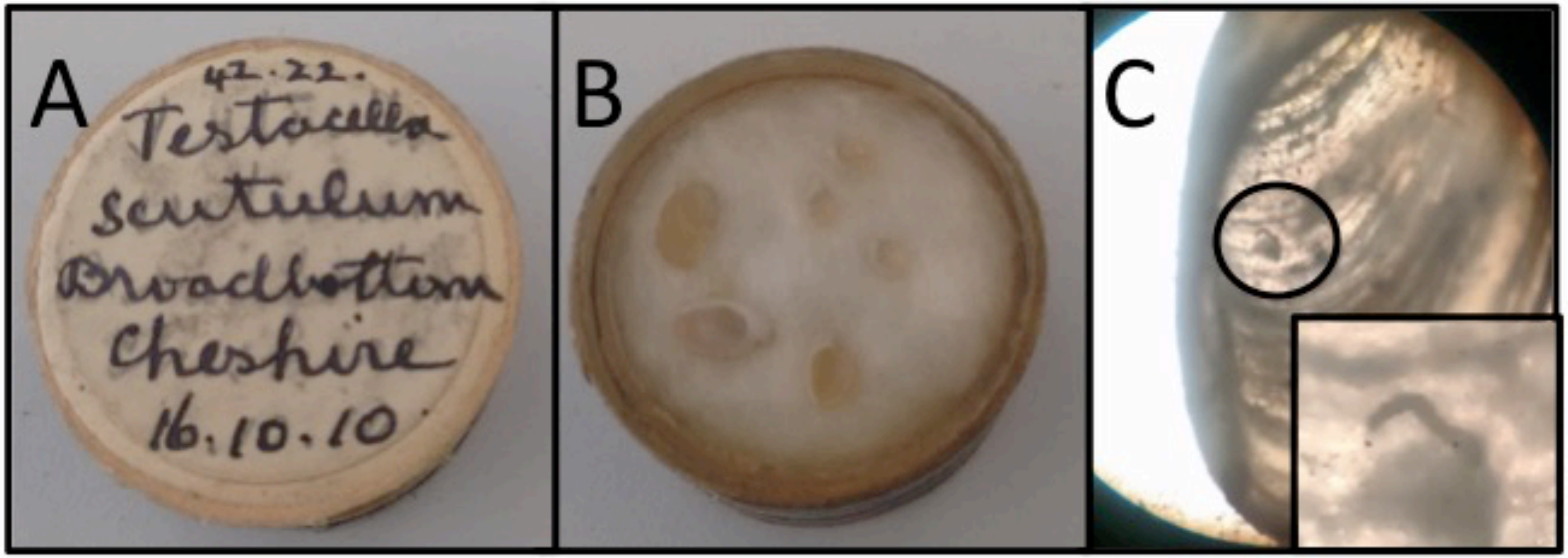

Testacella species	Number of shells examined	Number of shells with nematodes	Number of nematodes in shells (range)
Testacella scutulum	28	2	1 to 8
Testacella haliotidea	24	0	0
Testacella spp.	6	0	0
Testacella maugei	32	2	1
